# Orthopaedic Research Consortiums: A Review of Scope, Sex and Racial Representation

**DOI:** 10.7759/cureus.55859

**Published:** 2024-03-09

**Authors:** Yagiz Ozdag, A. Michael Luciani, Brian K Foster, Jessica L Baylor, Daniel S Hayes, Stephanie Gabelus, Louis C Grandizio

**Affiliations:** 1 Orthopaedic Surgery, Geisinger Medical Center, Danville, USA

**Keywords:** fellowship, publication, race, gender, orthopaedic research, research consortiums

## Abstract

Introduction

The creation of research groups and consortiums has become more common in all medical and surgical specialities. The purpose of this investigation was to assess and describe collaborative research groups and consortiums within orthopaedic surgery. In addition, we aimed to define the demographics of the research consortium members with particular attention to female and minority members.

Methods

Journals with a musculoskeletal/orthopaedic focus and a few medical journals were selected to identify articles published by research groups and consortiums. Articles published from 2020 to 2022 were manually reviewed. Bibliographic information, author information and level of evidence (LOE) were recorded. For identified consortium members, sex and race were defined in a binary manner.

Results

A total of 92 research consortiums were identified. A list of members was identified for 77 groups (83.7%), totalling 2,260 researchers. The remaining group members were not able to be identified due to the lack of information in the included publications, research group websites or after communicating with the corresponding author for respective articles. Most researchers were male (n=1,748, 77.3%) and white (n=1,694, 75%). Orthopaedic surgeons comprised 1,613 (71.4%) identified researchers. The most common fellowship training for orthopaedic surgeons was paediatrics (n=370, 16.4%), trauma (n=266, 11.8%) and sports medicine (n=229, 10.1%). The consortiums published 261 articles: women were lead (first) authors in 23% and senior (last) authors in 11.1%. Non-white researchers were lead authors in 24.5% (n=64) and senior authors in 17.2% (n=45). The most common level of evidence was level 3, accounting for 45.6% (n=119) of all publications. Level 1 evidence accounted for 12.6% (n=33) of published articles.

Discussion

Representation of women in orthopaedic research consortiums exceeds their representation in almost every orthopaedic professional society. There is less publicly available data to compare the involvement of under-represented minorities (URMs) in research consortiums to general practice. Further investigations should analyse possible avenues in which gender and racial disparity could be improved within orthopaedic surgery research.

## Introduction

The creation of research groups and consortiums has become more common in all medical and surgical specialities. These groups often function to align research interests and resources to produce large-scale, high-quality investigations. Incorporating multiple surgeons at high-volume centres can allow for adequate sample sizes to conduct prospective, randomized clinical trials or larger clinical series of rarer clinical entities. With collaboration between higher- and lower-income countries, research consortiums may also help establish sustainable services in lower-income countries [1,2]. Within orthopaedic surgery, the Multicenter Orthopaedic Outcomes Network (MOON) group remains among the most well-known. Originally consisting of three surgeons from two academic centres, the MOON group has expanded to include 19 surgeons at seven institutions since being founded in 2002 [3]. At present, this group has enrolled over 4,400 anterior cruciate ligament (ACL) reconstructions into a large prospective database and has conducted multiple level I studies that have been incorporated into clinical practice guidelines [4]. Similar collaborative efforts from other groups have resulted in large, clinically impactful randomized controlled trials (RCTs) [5,6]. Given the potential impact of collaborative consortiums, there may be opportunities for grant funding, academic advancement and career development for involved collaborators.

Although publications from research groups and consortiums continue to emerge, little is known regarding the origins of these groups. Like-minded colleagues from different academic centres may elect to formally collaborate to answer questions that require large sample sizes; however, the organization, requirements for participation and group structure often remain unclear. During the time that these groups have proliferated in the orthopaedic community, there has been increased recognition of the substantial lack of diversity in orthopaedics [7]. For example, multiple prior investigations have found that women are under-represented in podium presentations, leadership roles and as lead/senior authors in peer-reviewed publications [8-11].

Recently, major orthopaedic professional organizations have recognized the concerning lack of diversity in the field and have made formalized, structured efforts to enhance diversity, equity and inclusion. In 2019, the American Academy of Orthopaedic Surgeons (AAOS) created a five-year strategic plan aimed at increasing diversity among members and within leadership positions [12]. There have also been efforts from multiple subspecialty societies including the American Association of Hip and Knee Surgeons (AAHKS), the American Society for Surgery of the Hand (ASSH) and the North American Spine Society (NASS) [13-15]. These formal initiatives have appeared to have an early effect on enhancing diversity within orthopaedic surgery [9,11,16,17]. However, many research groups and consortiums exist outside of professional society oversight, and the demographics of group members remain uncertain.

The purpose of this investigation was to assess and describe collaborative research groups and consortiums within orthopaedic surgery. Research consortiums were chosen as the focus of the study, as there is no prior research dedicated to defining these groups in orthopaedics. In addition, we aimed to define the demographics of the research consortium members with particular attention to female and under-represented minority (URM) members. We hypothesized that female and URM involvement in these consortiums would be about equal to their representation in overall orthopaedic practice.

## Materials and methods

Institutional review board approval was not required for this study, which did not involve human subjects and utilized publicly available data. We defined research consortiums as named research groups with multiple members. There were no restrictions on the number of involved institutions. Journals were identified using the Scimago Journal Rankings where the included journals were selected via using the “Medicine” and “Orthopaedics and Sports Medicine” subject areas. No limitations were used for region/country selection to capture the widest possible selection of journals. High-impact medical and orthopaedic surgery journals that were not initially identified by this search were also manually selected to identify articles published by research consortiums. These journals were selected to cover general orthopaedic journals with the highest impact factors and the most prominent journal for each orthopaedic subspeciality as well as medical journals where high-impact articles related to orthopaedics are published. A list of the included journals can be found in Table 1.

**Table 1:** List of included journals

#	Journal Name(s)
1	Acta Ortopedica Brasileira
2	American Journal of Sports Medicine
3	Archives of Physical Medicine and Rehabilitation
4	Arthroplasty
5	Arthroscopy
6	Arthroscopy, Sports Medicine and Rehabilitation
7	Bone & Joint
8	Bone & Joint Open
9	British Medical Journal Open
10	Clinical Orthopaedics and Related Research
11	European Federation of National Associations of Orthopaedics and Traumatology Open Reviews
12	European Spine Journal
13	Foot and Ankle International
14	Foot and Ankle Surgery
15	Gait Posture
16	Journal of Bone & Joint Surgery
17	Journal of Foot and Ankle Research
18	Journal of Hand Surgery
19	Journal of Orthopaedic Trauma
20	Journal of Pediatric Orthopaedics
21	Journal of Shoulder & Elbow Surgery
22	Journal of the American Academy of Orthopaedic Surgeons
23	Journal of the American Academy of Orthopaedic Surgeons Global Research and Reviews
24	Journal of the American Medical Association
25	Journal of the American Medical Association Surgery
26	New England Journal of Medicine
27	Orthopaedic Journal of Sports Medicine
28	Spine
29	Spine Deformity

Articles published in each journal between January 2020 and December 2022 were manually reviewed, and articles produced by research groups or consortiums were recorded by six reviewers (Y.O, A.M.L, J.L.B., S.G, D.H.S, B.K.F). This period was utilized to identify recently active research consortiums. For each article, the author list, acknowledgements and abstracts were reviewed to identify any mention of a research group or consortium. Bibliographic information, including lead (first) and last (senior) authors, was recorded. The level of evidence of each article was independently graded using the definition described by Marx et al. [18]. For each group identified, an internet-based search was performed to identify a list of active members. Only information available through an official group or academic website was included. For groups whose member lists could not be obtained from the article or the website, the corresponding author of the identified article was contacted requesting membership information.

Once member lists were compiled, we recorded basic demographic information including race and sex in binary categories (white vs. non-white for race and male vs. female for sex) using a previously defined methodology [16,17]. For instances in which sex could not be determined manually, “gender-api” was used which has previously been shown to be >97% accurate in identifying sex based on names [19,20]. If race or sex could not be defined, no distinction was made. Institutional affiliation and fellowship training were also recorded for each identified consortium member. 

Statistical analysis

Descriptive statistics were utilized for this study. Categorical data was reported as count with frequency. A Chi-squared test (or Fisher’s Exact test, where appropriate) was conducted for categorical data, and student t-test was used for continuous data, where applicable. All statistical analysis was conducted using SPSS version 28.0.0.0 (IBM Corp., Armonk, NY).

## Results

A total of 92 research consortiums were identified. A list of the consortiums can be found in Table 2.

**Table 2:** List of all the identified research consortiums

#	Consortium Name
1	ACHE Study Group
2	ACTUAR Study Group
3	American Hip Institute Research Foundation
4	American Shoulder and Elbow Surgeons (ASES) Periprosthetic Joint Infection (PJI) Multicenter Group
5	ANCHOR Study Group
6	ASES B2 Glenoid Multicenter Research Group
7	ASES Complications of RSA Research Group
8	BASEL-PMI-Ortho Research Group
9	BC (BioCartilage) Study Group
10	BEST-Knee Study Team
11	Biologics Association
12	BITE Study Group
13	BOOM (Behavior in Orthopaedics Over Mental Health) Group
14	British Orthopaedic Oncology Society VTE Committee
15	British Orthopaedic Surgery Surveillance Study
16	Canadian Orthopaedic Trauma Society (COTS)
17	Canadian Spine Outcomes Research Network
18	CARE Consortium
19	Cleveland Clinic OME Arthroplasty Group
20	Cleveland Clinic Shoulder Group
21	Collaborative Orthopaedic Educational Research Group
22	CORE Research Group
23	CORE-Kids Collaborative Group
24	Department of Defense (DoD) Peer-Reviewed Orthopaedic Research Program (PRORP)
25	Dutch Clinical Spine Research Group
26	EF3X-trial Study Group
27	ESCMID Study Group for Implant-Associated Infections (ESGIAI)
28	Exeter Hip Group
29	FACTS (Function After Adolescent Clavicle Trauma and Surgery) Study Group
30	FISH Investigators
31	Fracture-related Infection (FRI) Consensus Group
32	Global Fragility Fracture Network Hip Fracture Audit Special Interest Group
33	Hamilton Arthroplasty Group
34	Hand-Wrist Study Group
35	Harms Study Group
36	Hip Society Research Group
37	HSS Orthopaedic Foot and Ankle Surgery Group
38	IMPACT Collaboration
39	International Ankle Arthroplasty Registry Consortium
40	International IPD-SMT Group
41	International Perthes Study Group
42	International Spine Study Group
43	Japan Spinal Deformity Institute Study Group
44	Keio Spine Research Group
45	Knee Arthroplasty Workgroup
46	LNAZ Research Group
47	LOAS Study Group
48	Lundbeck Foundation Centre for Fast-track Hip and Knee Replacement Collaborative Group
49	Machine Learning Consortium
50	Major Extremity Trauma Research Consortium (METRC)
51	MARS Group
52	McGill Scoliosis and Spine Research Group
53	Moon Knee Group
54	MRAB Study Group
55	MUKA Study Group
56	NORDSTEN-DS Investigators
57	Orthopaedic Electronic Learning Graduate Medical Education Consensus Working Group
58	Ottawa Arthroplasty Group
59	Oxford Upper Limb Collaborative
60	PAT Trial Collaborators
61	Pathologic Fracture Study Group
62	Pediatric Orthopaedic Obesity Research Consortium
63	Pediatric Research in Sports Medicine (PRiSM) Research Interest Group
64	Pediatric Spine Study Group
65	POSNA Evidence Based Committee
66	PRAISE-2 Investigators
67	Progressive Collapsing Foot Deformity Consensus Group
68	PRONOMOS Investigators
69	REGAIN Investigators
70	Regional Prosthetic Joint Infection Working Group
71	Revision Knee Replacement Priority Setting Partnership Steering Group
72	ROCK (Research in OsteoChondritis Dissecans of the Knee) Group
73	RODEO Collaborator Group
74	ROSA Study Group
75	SAPF (Swedish Arthroplasty for Proximal humeral Fracture) Study Group
76	SAPF Study Group
77	Science of Variation Group
78	SENSOR BALANCE Study Group
79	Spine Research Group
80	STABILITY Study Group
81	The Combined Randomised and Observational Study of Surgery for Fractures in the Distal Radius in the Elderly (CROSSFIRE) Study Group
82	The Knee Society
83	The MOON Shoulder Group (Multicenter Orthopaedic Outcomes Network)
84	The Pediatric ACL: Understanding Treatment Options (PLUTO Group)
85	Tibial Spine Fracture Research Interest Group
86	UK Infinity Study Group
87	VOICES Health Policy Research Investigators
88	Weight Bearing CT International Study Group
89	WHiST Trial
90	WHiTE 5 Investigators
91	WhiTE Collaborators
92	WHiTE Four Investigators

A list of members was identified for 77 groups (83.7%). In total, 2,260 researchers were identified. Most researchers were male (n=1,748, 77.3%) and white (n=1,694, 75%). Orthopaedic surgeons comprised 1,613 (71.4%) identified researchers. Fellowship training for orthopaedic surgeons was widely distributed with no single group comprising a substantial percentage. Fellowship information of the identified surgeons is displayed in Table 3.

**Table 3:** Fellowship and background information of the identified orthopaedic surgeons in the study

Fellowship/Background for Orthopaedic Surgeons	N (%)
Pediatric Orthopaedics	370 (16.4%)
Orthopaedic Trauma	266 (11.8%)
Sports Medicine	229 (10.1%)
Spine Surgery	191 (8.5%)
Adult Reconstruction	160 (7.1%)
Shoulder and Elbow Surgery	74 (3.3%)
Foot and Ankle Surgery	54 (2.4%)
Hand Surgery	45 (2%)
Orthopaedic Oncology	16 (0.7%)
General Orthopaedics	55 (2.4%)
Multiple Fellowships	120 (6.8%)

The identified research consortiums published 261 articles during the study period; bibliographic information of these studies including the level of evidence, publication topic, journal of publication and institutions representing the most members is included in Table 4. Overall, women were the lead authors in 23% (n=60) and senior authors in 11.1% (n=29) of publications. Demographic information of the identified authors can be found in Table 5.

**Table 4:** Description and demographics of included orthopaedic research groups/consortiums and published articles *Percent of total identified members. +Percent of publications.

Research Group/Consortium Demographics	
Total groups identified, n	92
Groups with identified members, n (%)	77 (83.7%)
Single centre groups	10 (13%)
Dual or Multi-centre groups	67 (87%)
Total members identified, n (%)	2260
Median number of members (range)	18 (5-222)
Publication demographics	
Total publications identified, n	261
Mean publications per group, mean (SD)	2.8 (8.3)
Level of Evidence for Identified Publications	n (%)
Level I	33 (12.6%)
Level II	46 (17.6%)
Level III	119 (45.6%)
Level IV	37 (14.2%)
Level V	20 (7.7%)
N/A	6 (2.3%)
Article Type	n (%)
Clinical Investigation	254 (92.7%)
Basic Science Investigation	7 (2.7%)
Publication topic	n (%)
Spine	105 (40.2%)
Trauma	41 (15.7%)
Adult Reconstruction	27 (10.3%)
Sports	20 (7.7%)
Shoulder and Elbow	18 (6.9%)
Pediatric Orthopaedics	16 (6.1%)
Foot and Ankle	12 (4.6%)
Other	10 (3.8%)
Hand	7 (2.7%)
General Orthopaedics	4 (1.5%)
Orthopaedic Oncology	1 (0.6%)
Top 5 institutions*	n(%)
1. Cleveland Clinic	58 (2.6%)
2. Boston Children's Hospital	45 (2%)
3. Children’s Hospital of Philadelphia	30 (1.3%)
4. Shriner’s Children	26 (1.2%)
5. Hospital for Special Surgery	24 (1.1%)
Top 5 Journals of Publication^+^	n(%)
1. Spine	50 (19.2%)
2. Journal of Pediatric Orthopaedics	31 (11.9%)
3. Bone and Joint Journal	27 (10.3%)
4. Spine Deformity	22 (8.4%)
5. The American Journal of Sports Medicine	21 (8%)

**Table 5:** Demographics for identified authors/collaborators within orthopaedic research groups/consortiums

Total groups identified, n	92
Groups with identified members, n (%)	77 (83.7%)
Single centre groups, n (%)	10 (13%)
Dual or multi-centre groups, n (%)	67 (87%)
Total members identified, n (%)	2260
Median number of members (range)	18 (5-222)
Researcher sex	n (%)
Male	1748 (77.3%)
Female	398 (17.6%)
Unknown	114 (5%)
Sex of lead author	n (%)
Male	195 (74.7%)
Female	60 (23%)
Unknown	6 (2.3%)
Race of lead author	n (%)
White	187 (71.6%)
Non-white	64 (24.5%)
Unknown	10 (3.8%)
Sex of senior author	n (%)
Male	225 (86.2%)
Female	29 (11.1%)
Unknown	7 (2.7%)
Race of senior author	n (%)
White	211 (80.8%)
Non-white	29 (11.1%)
Unknown	5 (1.9%)
Researcher race	n (%)
White	1694 (75%)
Non-white	368 (16.3%)
Unknown	198 (8.8%)
Clinical background	n (%)
Orthopaedic surgeon	1613 (71.4%)
Physician Researcher, non-surgeon (MD/DO)	142 (6.3%)
Surgeon researcher, non-ortho	39 (1.7%)
Non-physician researcher	316 (14%)
Unknown	150 (6.6%)

Non-white researchers were lead authors in 24.5% (n=64) and senior authors in 17.2% (n=45) of publications. The most common level of evidence was level 3, accounting for 45.6% (n=119) of all publications (Table 4). Level 1 evidence accounted for 12.6% (n=33) of published articles. The most common subspecialty topics were spine (n=105, 40.2%), trauma (n=41, 15.7%) and adult reconstruction (n=27, 10.3%) (Table 4). Comparisons between the percentage of female and URM members of the identified orthopaedic research consortiums to orthopaedic professional societies are shown in Table 6.

**Table 6:** Sex and racial demographics for major orthopaedic professional organizations

Orthopaedic Professional Society	% Female Members	% Non-White Members
American Academy of Orthopaedic Surgeons (AAOS)	5.8% [8]	14.1% [8]
American Society for Surgery of the Hand (ASSH)	15.6% [21]	17% [22]
American Orthopaedic Society for Sports Medicine (AOSSM)	9% [23]	-
North American Spine Society (NASS)	10% [23]	-
Orthopaedic Trauma Association (OTA)	14.1% [24]	
American Shoulder and Elbow Society (ASES)	6% [23]	-
Pediatric Orthopaedic Society of North America (POSNA)	24% [25]	15.9% [26]
American Orthopaedic Foot and Ankle Society (AOFAS)	13% [27]	-
American Association of Hip and Knee Surgeons (AAHKS)	3.1% [28]	-
Musculoskeletal Tumor Society (MTS)	11-14% [29]	-
Identified Orthopaedic Research Consortiums	17.6%	16.3%

## Discussion

In agreement with our hypothesis, when comparing female representation in orthopaedic research consortiums to that of general orthopaedic practice, their involvement appears more favourable. Our data indicated that 18% of identified consortium members were female. According to the 2018 AAOS Census, only 6% of practising orthopaedic surgeons are female, with 14% identifying as a racial minority [7]. While women in orthopaedics remain under-represented relative to the proportion of women in medical school and the general population, our results indicate that the representation of female surgeons in orthopaedic consortiums exceeds their representation in almost every orthopaedic professional society [21,24,25,27,28]. Similarly, women account for 18% of all faculty positions and 19% of orthopaedic society leadership positions, which also outpaces the recent AAOS census demographic breakdown [30,31]. It is possible the observed results of female involvement in orthopaedic research consortiums are inherently skewed, as members of these research consortiums are also likely to be involved in academic practices and there is a greater percentage of women in academic positions compared to general orthopaedic practice.

Considering the percentage of women serving as lead authors in the studies produced by consortiums (23%), female surgeons are not only achieving higher levels of representation within research groups, but they are also more frequently functioning in primary roles for these collaborative research projects. It should be noted that women accounted for only 11% of senior authors of studies. These results are not surprising, given the traditional progressions in authorship observed over the course of an academic career. It is likely that as women become more involved in these collaborative efforts (particularly in leadership roles), senior authorship may also increase with time. Hiller et al., in a review of leading orthopaedic journals over a 12-year period (2006-2017), reported that women were lead authors in 13% of articles and senior authors in 10% of articles [8]. Recent studies suggest females comprise only 16% of orthopaedic surgery residents, 4% of American Academy of Orthopaedic Surgery fellows and 6% of practising orthopaedic surgeons [7,11,32-34]. Our results suggest female authorship, and involvement in orthopaedic research consortiums appears to be greater than in the overall orthopaedic literature and general practice.

When examining the membership of URMs in orthopaedic research consortiums, this study found they comprised 368 (16.3%) members. This representation is similar to several orthopaedic professional societies [7,26]. However, this is a smaller percentage than what is reported in academic orthopaedics based on a recent study that investigated the demographic make-up within the board of directors, editorial boards, National Institute of Health (NIH) grant recipients and accreditation boards [35]. We found 24.5% of lead authors to be URMs, which appears favourable considering that this population comprised only 16.3% of members of the consortiums. URM authors were also well represented in terms of senior authorship, comprising 17.2% of senior authors in this study. It is unknown how these trends compare to general orthopaedic practice and research independent of research consortiums.

As stated previously, spine was by far the most common topic published by these orthopaedic research consortiums with about 40% of all publications being spine-related topics. Over twice as much as the next most common topics of trauma (n=41, 15.7%) and adult reconstruction (n=27, 10.3%). However, it is not completely clear to the authors why this was observed, as there is no prior research to the authors’ knowledge to suggest this. However, the authors can speculate that spine may have had more involvement due to many of the topics in spine overlapping other subspecialties of orthopaedics, in particular paediatrics, which was found to be the most common fellowship training of orthopaedic surgeons in consortiums. For example, many of the spine-related articles focused on scoliosis, as well as other paediatric syndromes with spine manifestations.

Peer-reviewed publications produced by modern orthopaedic research groups and consortiums exist in all orthopaedic subspecialities. The majority (87%) of these groups were collaborative, multi-centre efforts with a median of 18 members. In general, orthopaedic research consortiums appear to be publishing studies with higher LOE compared to levels noted in prior bibliometric studies of orthopaedic literature. The average LOE in orthopaedic research has historically been poor, with only 0.8% of articles published by 2002 and 4.1% by 2012 containing level I evidence [36]. We found 12.6% of identified investigations produced by research consortiums contained level I evidence and over 76% of studies contained at least level III evidence. Several prior studies have shown that level IV evidence comprised between 43% and 63% of their bibliometric analyses of orthopaedic studies [36-38]. A more recent study of orthopaedic literature from 2013 to 2018 found an increase in the mean level of evidence and suggested that level III evidence has become more prevalent, while the frequency of high-level studies (defined as level I or level II evidence) decreased over the same time period [39]. Conducting large clinical trials with appropriate sample sizes and study power may require collaboration and multi-centre involvement. These investigations can be logistically difficult, and many prospective clinical trials are terminated prior to completion [40]. In this context, research consortiums may be ideal avenues to design and implement these challenging clinical investigations.

Limitations

This study has several limitations. We utilized a three-year period for identifying research consortiums. Although this was done to exclude inactive consortiums that have not published recently, it is possible that we missed active groups that publish infrequently. In addition, we included a variety of journals; however, it is possible that there were consortiums that published in journals that were not included. We were unable to identify members for 15 groups (16.3%) and it is uncertain how the demographics of unidentified members would have impacted our results. We elected to define race and sex in binary terms by searching the internet based on previous methodologies; however, this type of classification is not without limitations. For example, we were unable to determine the sex of 114 (5%) researchers and unable to determine the race of 198 (8.8%) researchers. As a final limitation, our definition of URMs is based on the racial breakdown in the US and, therefore, may not be applicable to other nations.

## Conclusions

In conclusion, while women in orthopaedic research consortiums remain under-represented relative to the proportion of women in medical school, their representation in research consortiums exceeds their representation in almost every orthopaedic professional society. There is insufficient information to make any comparisons regarding the involvement of URMs in research consortiums to general orthopaedic practice. Orthopaedic research consortiums appear to be publishing studies with higher LOE relative to the LOE assessed in prior bibliometric studies of orthopaedic literature. We hope this work encourages further research on this topic.

## References

[1] Ritman D (2016). Health partnership research and the assessment of effectiveness. Global Health.

[2] Miclau T, MacKechnie MC, Shearer DW (2018). Consortium of orthopaedic academic traumatologists: a model for collaboration in orthopaedic surgery. J Orthop Trauma.

[3] (2023). MOON Knee Group: MOON Knee Group participating institutions and physicians. https://acltear.info/moon-knee-group/participating-institutions/.

[4] Lynch TS, Parker RD, Patel RM, Andrish JT, Spindler KP (2015). The impact of the Multicenter Orthopaedic Outcomes Network (MOON) research on anterior cruciate ligament reconstruction and orthopaedic practice. J Am Acad Orthop Surg.

[5] Chung KC, Kim HM, Malay S, Shauver MJ (2021). Comparison of 24-month outcomes after treatment for distal radius fracture: the WRIST randomized clinical trial. JAMA Netw Open.

[6] Lawson A, Naylor J, Buchbinder R (2020). A Combined Randomised and Observational Study of Surgery for Fractures In the distal Radius in the Elderly (CROSSFIRE): a statistical analysis plan. Trials.

[7] American Academy of Orthopedic Surgeons (2023). American Academy of Orthopedic Surgeons: Orthopedic practice in the United States, survey 2018. https://www.aaos.org/globalassets/quality-and-practice-resources/census/2018-census.pdf.

[8] Hiller KP, Boulos A, Tran MM, Cruz AI Jr (2020). What are the rates and trends of women authors in three high-impact orthopaedic journals from 2006-2017?. Clin Orthop Relat Res.

[9] Tougas C, Valtanen R, Bajwa A, Beck JJ (2020). Gender of presenters at orthopaedic meetings reflects gender diversity of society membership. J Orthop.

[10] Brisbin AK, Chen W, Goldschmidt E, Smith BT, Bourne DA (2023). Gender diversity in hand surgery leadership. Hand (N Y).

[11] Nwosu C, Wittstein JR, Erickson MM (2023). Representation of female speakers at the American Academy of Orthopaedic Surgeons Annual Meetings Over Time. J Am Acad Orthop Surg.

[12] (2024). American Academy of Orthopaedic Surgeons: AAOS 5-year strategic plan. https://www.aaos.org/about/meet-aaos/aaos-strategic-plan/.

[13] (2023). American Society for the Surgery of the Hand (ASSH): ASSH Policies. https://www.assh.org/s/policies.

[14] (2023). American Association of Hip and Knee Surgeons: AAHKS Gender Disparity in Orthopaedic Surgery. https://hipknee.aahks.org/gender-diversity-in-orthopaedic-surgery.

[15] (2023). American Medical Women's Association: AMWA and the North American Spine Society (NASS). https://www.amwa-doc.org/news/amwa-and-the-north-american-spine-society-nass/.

[16] Grandizio LC, Pavis EJ, Hayes DS, Young A, Klena JC (2021). Analysis of gender diversity within hand surgery fellowship programs. J Hand Surg Am.

[17] Ozdag Y, Baylor JL, Delma S, El Koussaify J, Zelenski NA, Grandizio LC (2023). An analysis of gender diversity in hand and upper extremity surgery webinars. J Hand Surg Am.

[18] Marx RG, Wilson SM, Swiontkowski MF (2015). Updating the assignment of levels of evidence. J Bone Joint Surg Am.

[19] (2022). Gender-API. http://gender-api.com.

[20] Sebo P (2021). Performance of gender detection tools: a comparative study of name-to-gender inference services. J Med Libr Assoc.

[21] Letchinger R, Kerluku J, Wessel LE, Noland S, Fufa DT (2022). Assessing and addressing gender gaps in the American Society for Surgery of the Hand. J Hand Surg Am.

[22] Earp BE, Mora AN, Rozental TD (2018). Extending a hand: increasing diversity at the American Society for Surgery of the Hand. J Hand Surg Am.

[23] Gerull KM, Kim DJ, Cogsil T, Rhea L, Cipriano C (2020). Are women proportionately represented as speakers at orthopaedic surgery annual meetings? A cross-sectional analysis. Clin Orthop Relat Res.

[24] Murphy L, Miller AN, Vallier HA, Roffey DM, Lefaivre KA (2023). Gender diversity, leadership, promotion, and opportunity among the members of the Orthopaedic Trauma Association (OTA). J Orthop Trauma.

[25] (2023). Pediatric Orthopaedic Society of North America (POSNA): The Pediatric Orthopaedic Society of North America: where are we now?. https://posna.org/POSNA/media/Documents/Membership/2022_POSNA_History_1996_2021.pdf.

[26] Singleton IM, Poon SC, Bisht RU, Vij N, Lucio F, Belthur MV (2021). Diversity and inclusion in an Orthopaedic Surgical Society: a longitudinal study. J Pediatr Orthop.

[27] Chrea B, Johnson H, Baumhauer J, Holleran A, Atwater LC, Poon S (2022). A 10-year review of designated leadership positions of the American Orthopaedic Foot & Ankle Society (AOFAS). Foot Ankle Orthop.

[28] Cohen-Rosenblum AR, Bernstein JA, Cipriano CA (2021). Gender representation in speaking roles at the American Association of Hip and Knee Surgeons Annual Meeting: 2012-2019. J Arthroplasty.

[29] Martinez M, Lopez S, Beebe K (2015). Gender comparison of scholarly production in the Musculoskeletal Tumor Society Using the Hirsch Index. J Surg Educ.

[30] Chambers CC, Ihnow SB, Monroe EJ, Suleiman LI (2018). Women in orthopaedic surgery: population trends in trainees and practicing surgeons. J Bone Joint Surg Am.

[31] Albright P, Banks E, Wood L, Chambers C, Van Heest A (2022). Orthopaedic Society Leadership Diversity and Academic Participation: where do we stand now?. J Bone Joint Surg Am.

[32] Van Heest AE, Agel J (2012). The uneven distribution of women in orthopaedic surgery resident training programs in the United States. J Bone Joint Surg Am.

[33] (2023). Association of American Medical Colleges (AAMC): Active physicians by sex and specialty, 2019. https://www.aamc.org/data-reports/workforce/interactive-data/active-physicians-sex-and-specialty-2019.

[34] Lattanza LL, Meszaros-Dearolf L, O'Connor MI, Ladd A, Bucha A, Trauth-Nare A, Buckley JM (2016). The Perry initiative's Medical Student Outreach Program recruits women into orthopaedic residency. Clin Orthop Relat Res.

[35] Vij N, Singleton I, Bisht R, Lucio F, Poon S, Belthur MV (2022). Ethnic and sex diversity in academic orthopaedic surgery: a cross-sectional study. J Am Acad Orthop Surg Glob Res Rev.

[36] Little Z, Newman S, Dodds A, Spicer D (2015). Increase in quality and quantity of orthopaedic studies from 2002 to 2012. J Orthop Surg (Hong Kong).

[37] Murray MR, Wang T, Schroeder GD, Hsu WK (2012). The 100 most cited spine articles. Eur Spine J.

[38] Holzer LA, Holzer G (2014). The 50 highest cited papers in hip and knee arthroplasty. J Arthroplasty.

[39] Luksameearunothai K, Chaudhry Y, Thamyongkit S, Jia X, Hasenboehler EA (2020). Assessing the level of evidence in the orthopaedic literature, 2013-2018: a review of 3449 articles in leading orthopaedic journals. Patient Saf Surg.

[40] Caruana DL, Gouzoulis MJ, McLaughlin WM, Grauer JN (2022). Analysis of the frequency, characteristics, and reasons for termination of shoulder- and elbow-related clinical trials. J Shoulder Elbow Surg.

